# A century of change in global education variability and gender differences in education

**DOI:** 10.1371/journal.pone.0212692

**Published:** 2019-02-27

**Authors:** Iñaki Permanyer, Diederik Boertien

**Affiliations:** Centre d’Estudis Demogràfics (a member of the CERCA Programme / Generalitat de Catalunya), Cerdanyola del Vallès, Barcelona, Spain; Aga Khan University, KENYA

## Abstract

The aim of this article is to document and understand how trends in educational variability and the gender gap in education developed jointly over time. Main questions include: Is the education distribution among women becoming more dispersed as their average attainment surpasses that of men? Is education variability among women higher than that of men? Does the reduction–and eventual reversal–of the gender gap in education go hand-in-hand with less educational variability overall? To answer these questions we first show how overall education variability can be decomposed into four clearly interpretable components (variability among women and men, educational advantage favoring women and favoring men). We subsequently document how these components have evolved over time in the world and its regions from 1950 to 2010 (with projections until 2040). Our findings suggest that (i) with education expansion, education variability tends to follow an inverted U-shape trajectory. (ii) The composition of education variability has been shifting dramatically over time; in particular (iii) variability among men was usually higher than variability among women until the turn of the millennium, from then onwards their educational attainment distributions have the same degree of dispersion. And (iv) while in the 1950s the educational advantage of men was by far the main contributor to education variability, nowadays the educational advantage of women has become the most important source of variability in high- and middle-income countries.

## Introduction

For a long time, a global expansion of mass schooling has been sweeping the globe. This process, which has been observed in all countries around the world, has been highly beneficial for an ever-growing number of individuals, so this is a worthy cause for celebration [1]. While an expanding body of scholarship has shed considerable light on the ‘efficiency part’ of the process (i.e. the country-level average education attainments are reasonably well-documented), much less is known about the ways in which education is distributed across individuals. With education expansion, it is likely that some sectors of the population benefit disproportionately more than others, thus generating increasingly disperse education distributions. Inequality in education is a matter of great scientific interest and policy concern that, unfortunately, has not received much scholarly attention and is still quite poorly understood.

One of the key characteristics of the education distribution that has attracted the attention of many scholars is the gender gap in education. During the last decades we are witnessing the emergence of a “global first” phenomenon: the closing and reversal of the gender gap in educational attainment. For the first time in history young women are attaining higher levels of education than young men in many high- and middle-income countries, and the same trend is expected to occur in other countries as well in the coming decades [2]. While relevant and informative, the comparisons of average attainments for women and men typically ignore the potential heterogeneity that might exist within those groups. Yet, implicitly treating women and men as if they were homogeneous groups does not allow addressing key questions about the education distribution. For instance: Is the education distribution among women becoming more dispersed as their average attainment surpasses that of men? Is education variability among women higher than that of men? Does the reduction–and eventual reversal–of the gender gap in education go hand-in-hand with less educational variability overall? How are each of these concepts related with one another? While it seems clear that (i) overall education variability, (ii) education variability among women and among men, and (iii) educational advantage of men over women and of women over men are interrelated with one another, no previous study has attempted to investigate and flesh out the nature of that relationship. For the first time, in this paper we put together these different ingredients into a coherent whole to explore how overall education variability is decomposed and relates to its basic constituent parts.

The relevance of documenting the trends in these different distribution-related indicators stems from their differential impacts on societies around the world, which are multiple and might go in opposite directions. On the one hand, increasing education variability–either for the population as a whole or for women and men separately–serves as a wellspring for increases in inequality in many other quality of life domains (higher variability in educational attainment is associated with higher dispersion in income and wages, higher job insecurity, and lower economic growth, occupational mobility, physical and mental health and general well-being–see [3–4]). On the other hand, the closing and reversal of the gender gap in education in favor of women might increase female labor force participation (particularly in better-paying formal-sector jobs), prolong the duration of women in the labor force (which in turn could lead to more efficient economic outcomes) [5] and generate more egalitarian attitudes (both in the domestic and public spheres [2]), that could eventually shift the balance of power towards females [6]. Symmetrically, high levels of gender inequality in favor of men have long been considered to be detrimental for the economic performance of countries and the well-being of their inhabitants [5]. For these reasons, it becomes important to explore whether and to what extent education variability and the gender gap in education move in the same or opposite directions–an issue that, to the best of our knowledge, has not yet been investigated.

Summing up, this article aims to explore how overall education variability is articulated in different moving parts and how these parts have jointly evolved over time in the world and its regions. Such decomposition is extremely useful for scholars and policy-makers to go beyond purely descriptive results and to analyze what factors are the most important drivers of education variability and its evolution over time. To attain our research objectives, we have created a new measure of education variability specifically crafted to simultaneously meet two requirements that are not met by currently existing measures. First, our measure nicely decomposes overall variability in two broad components: variability within groups (including variability among women and among men) and between groups (which can be further decomposed in two subcomponents: the educational advantage of men over women and of women over men). Second, it has been designed to handle the ordinal data we are dealing with in this paper (currently, virtually all education variability measures are based on cardinal information, like ‘years of schooling’). Hopefully, the new measure proposed in this paper can be a useful addition to those practitioners aiming at gauging variability in the context of ordinal variables.

The empirical analysis relies on the latest version of the Barro and Lee [7] database (henceforth BL) for the period from 1950 to 2010, as well as on some of our own projections, extending the results up to 2040. Such projections are solely intended to glimpse at what might be the evolution of education variability in case current trends were to continue during the coming decades following a simple logistic growth curve. The huge geographic coverage of the database (146 countries) allows performing both global and regional analyses over time.

## Background and hypotheses

It is difficult to overestimate the importance of education for the well-being of individuals across their entire life cycle. In general, highly educated individuals tend to have a better command over resources, higher levels of employment and better-paid jobs, they have longer and less unequal lifespans, experience lower risks of getting divorced, of being poor or becoming materially deprived and enjoy higher levels of subjective well-being [8–12]. Indeed, education is a key stratification variable in demographic behavior [13]. For these reasons, the education expansion that has been sweeping the world during the last decades is a phenomenon that a priori should be highly advantageous for those who benefit from it. This expansion includes rising literacy rates [14] as well as increases in schooling enrollment rates and in completed years of primary, secondary and college education [1, 7,15–19]. Regarding college education, by 1970, 6.4% of the world’s population aged 25–29 had obtained a college degree. Three decades later, this proportion had increased to 13%, and the expected figure for 2050 is 29.4% [20].

What do we know about global, regional and national trends in education variability and the gender gap in education? Despite its relevance for many socio-demographic and economic outcomes–e.g. high levels of education variability are associated with higher economic dispersion and unemployment levels, lower social cohesion and higher poverty rates [4]–researchers have only recently started to study how individuals’ education is distributed across and within countries (e.g. [1, 3, 21–25]). While these studies differ in many respects (e.g. they typically employ different indicators, datasets and methodological approaches, and/or their geographic and temporal coverage does not necessarily coincide) they all cohere with the following narrative. As mass education started to expand–predominantly in favor of men–education variability began to increase. After several decades, as education expansion gradually shifted the population from low to highly educated categories, education dispersion reached its highest point and afterwards started declining until the present day. In other words: empirical evidence seems to support the existence of a Kuznets curve in education. This highly simplified narrative generally applies for the world as a whole, its different regions and most of its countries (the studies by Castelló and Domenech [21], Benaabdelaali et al. [22], Meschi and Scervini [23] and Jordá and Alonso [25] focus on the second half of the 20th century, so they cover the downward part of the inverted U-shape trajectory; the studies by Dorius [3] and Morrisson and Murtin [1, 24] use data from the 19th to the 20th century, so they cover both the upward and downward portions of the trajectory).

A notable feature of the education expansion process is that it has not been gender neutral [6, 26–27]. Despite initially favoring males, the gender gap has closed rapidly in recent years and, in many countries, even reversed in favor of women [28], a trend that is expected to continue over the next decades [13, 20]. In 1970, men represented 63.6% of the total college educated population. This percentage decreased to 52.6% in 2000 and it is likely to reach 44% in 2050, with most high-income countries reaching lower levels [20]. The recent and prospective sex-specific share shifts across the education ladder–usually in favor of women–have attracted the attention of several researchers because of their potentially sizeable impacts across the board (e.g. fertility changes, higher female labor force participation and higher economic growth, or shifting power relations both in public and private domains; see [2, 28–30]). It should be pointed out that the gender gap in education–which is currently favoring women in most high- and middle-income countries–is not as large as it used to be when it favored men during the initial stages of the education expansion.

### Education variability and its components: Trends and hypotheses

**Fig 1:**
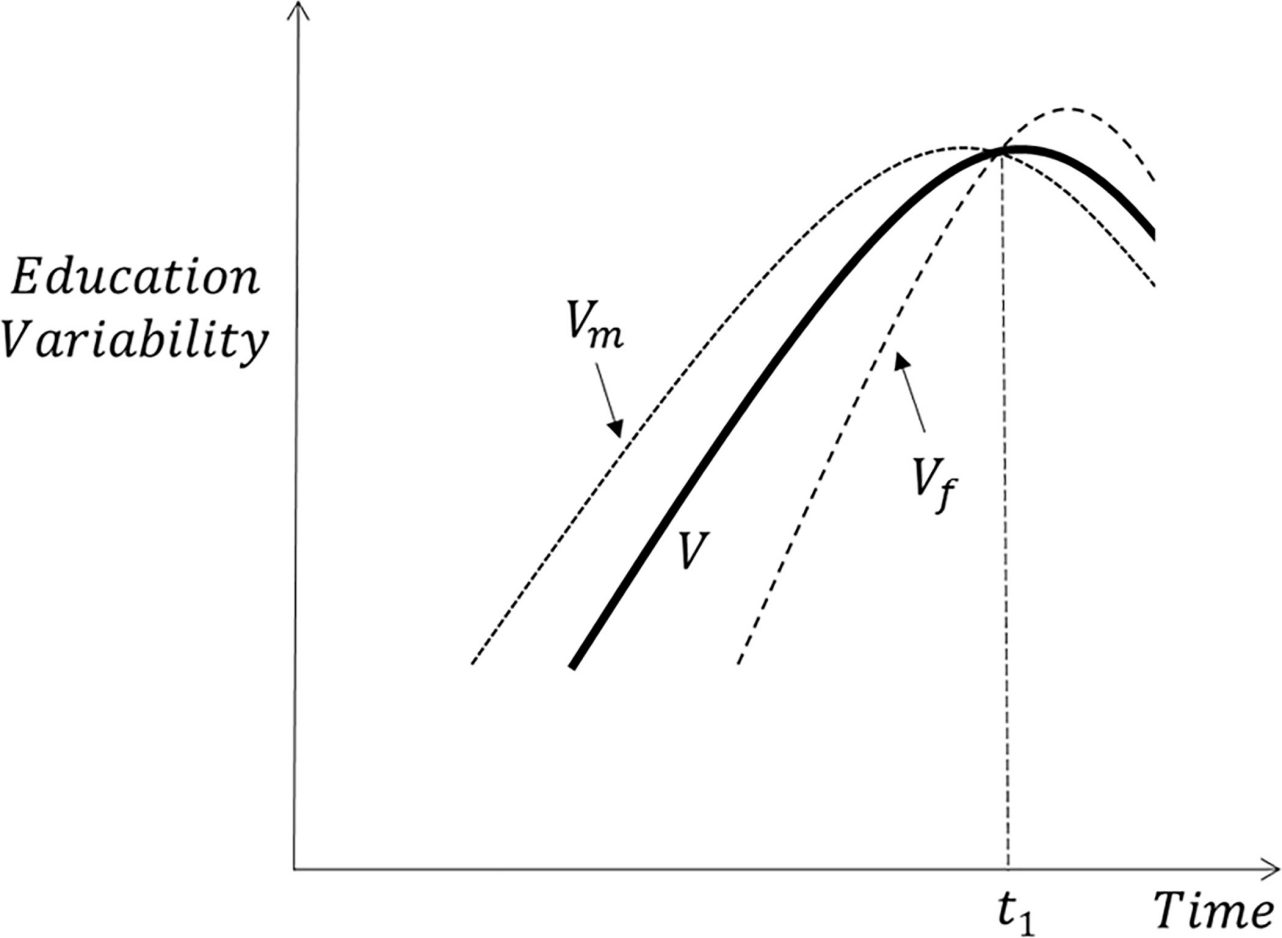
Schematic relationship between overall education variability (*V*) and variability among women (*V*_*f*_) and men (*V*_*m*_). Source: Authors’ elaboration.

Having scarce empirical evidence to rely upon, we cannot but hypothesize what might be the relationship between overall education variability (henceforth denoted as *V*) and its ‘basic subcomponents’: variability among men (*V*_*m*_), variability among women (*V*_*f*_), and the two components of the gender gap in education (i.e. educational advantage favoring men (*A*_*m*_) and educational advantage favoring women (*A*_*f*_) – details given in the methods section). A priori, there are many possible ways in which these subcomponents might have evolved over time in congruence with the aforementioned stylized narratives on the trends in overall education variability and the gender gap in education. In this regard, Fig 1 plots what might have been the hypothetical trajectories of *V*,*V*_*m*_ and *V*_*f*_ over time. In line with the existing empirical evidence, overall education variability is posited to first increase and then decrease. What about the education variability among men and among women? Since the education expansion initially benefited men, we expect *V*_*m*_ to increase earlier than *V*_*f*_. Yet, since the education expansion for women took place in a shorter period of time, we expect *V*_*f*_ to increase faster than *V*_*m*_. Based on the evidence reporting the closing and reversal of the gender gap in education in favor of women we expect *V*_*f*_ to exceed *V*_*m*_ at some point in time (denoted as *t*_1_ in Fig 1). Yet, given that it is unlikely that obstacles to access education, which women experienced in the past, will be put in place for men, we do not expect the differences in these two quantities to be as large as they might have been during the first stages of the education expansion that predominantly benefited men (i.e. we expect the two curves to remain relatively close to each other after time *t*_1_). Whether or not this has actually been the case for the different world regions will be investigated in the empirical section of the paper.

**Fig 2:**
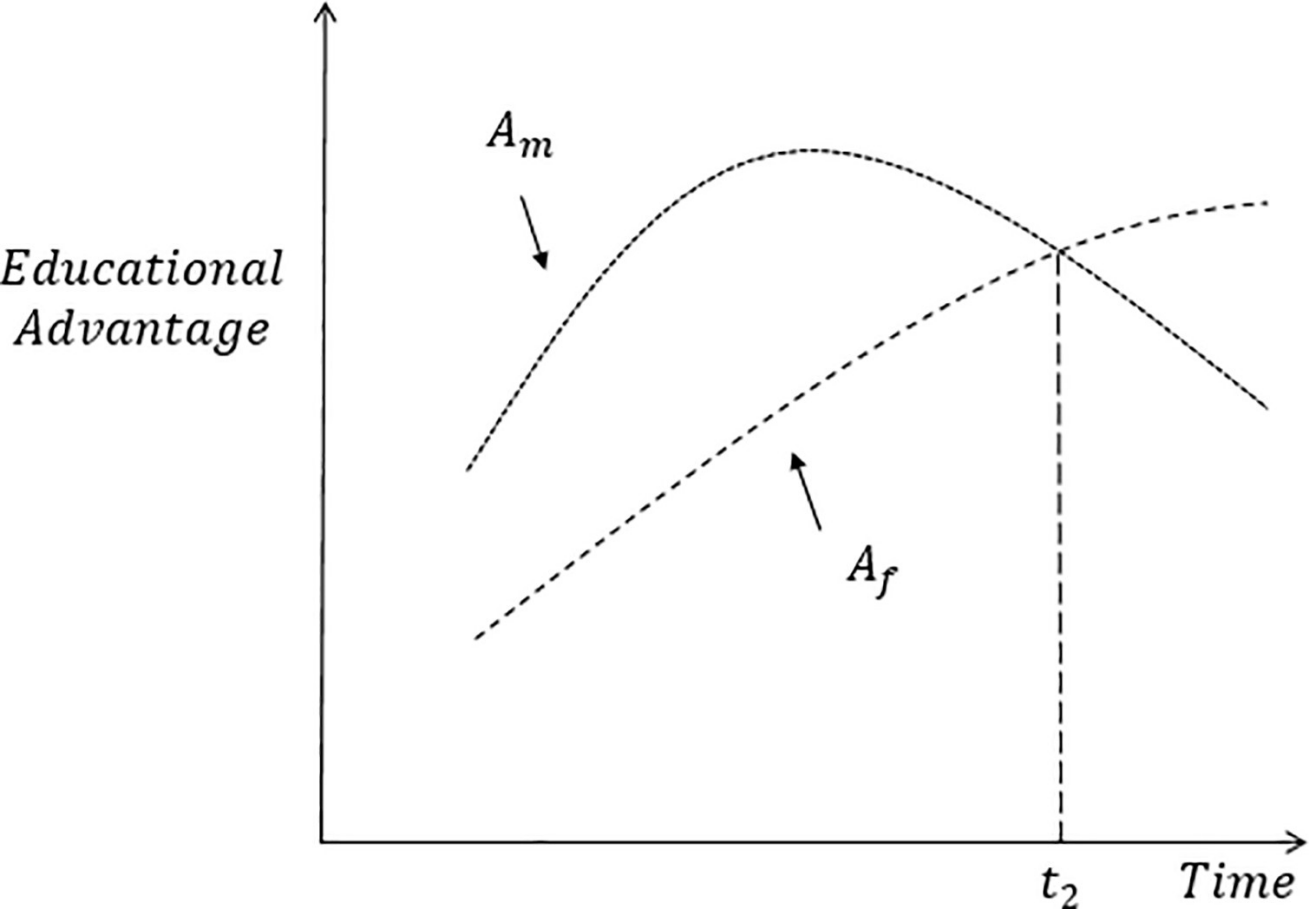
Schematic evolution of male and female educational advantage (*A*_*m*_, *A*_*f*_) over time. Source: Authors’ elaboration.

What about the two components of the gender gap in education? As shown in the methods section, *A*_*m*_ can be interpreted as the likelihood that men are more highly educated than women, and the opposite goes for *A*_*f*_. Based on current empirical evidence, we expect the educational advantage of men vis-à-vis women (*A*_*m*_) to follow an inverted U-shape, increasing first in the initial stages of education expansion and declining afterwards as the expansion starts benefiting women (see Fig 2). The same evidence suggests that, as an increasing proportion of women obtain higher education credentials than men, *A*_*f*_ should increase monotonically over time but with some delay with respect to the *A*_*m*_ curve. Depending on the relative position of these two curves (which is expected to vary across world regions), the gender gap in education (which can be defined as the difference between the previous two curves) might become smaller, eventually disappear or even reverse in favor of women. If the two curves eventually cross in some point in time (denoted as *t*_2_ in Fig 2), the corresponding gender gap reverses and changes its sign.

Lastly, since no previous studies have investigated the relationship between the trends in education variability and the gender gap in education, we have no clear expectations of how it might look like. A priori, both measures of variability might go in the same or in opposite directions, depending on the time period or the world region we are looking at. Essentially, it all boils down to determining the relative position of the different curves depicted in Figs 1 and 2, an issue that will be explored in the empirical section of the paper.

## Data

Like many studies on education inequality we have used the dataset of Barro and Lee [7], which is currently the largest in terms of geographic and temporal coverage. The data is based on a compilation of censuses and surveys by UNESCO and provides comparable information on seven educational attainment categories (no education, primary uncompleted, primary completed, secondary uncompleted, secondary completed, tertiary uncompleted and tertiary completed) for 146 countries during the period 1950–2010. Whenever appropriate, these categories can be reduced to four (no education, some primary, some secondary and some tertiary).

We used the estimates from Barro and Lee on educational attainment for the period 1950–2010 without further adjustments. At the same time, we decided to calculate our own projections of future educational attainment using a similar procedure to Barro and Lee’s. Calculating our own predictions allowed us to focus on the age group 30–34, instead of the age group 15–64 for which the projections of Barro and Lee were calculated. Selecting this age group enables us to minimize the influence of changes in the age structure within countries on our estimates and at the same time will show the most recent changes in educational attainment (by the age of 30–34, the vast majority of individuals have achieved their highest educational attainment). Another motivation has been that some implausible values were observed in the Barro and Lee projections (in some Western countries the attainment of tertiary education for the age group 25–64 was predicted to decline dramatically with time, such as Australia, Austria, and Finland, which have been resolved in our predictions). *STATA 14* was used for all data management and calculations made for this paper.

### Projections

Our projections of future educational attainment are based on logistic growth curve models estimated for the period 1950–2010 that allow for country-specific time trends (random slopes using STATA 14’s *xtmixed* package). The model, estimated separately for men, women, and each of the three educational stages, can be expressed as follows:

$$ln\left(\frac{s_{jt}}{100-s_{jt}}\right)=\alpha_{j}+\beta_{j}t+\mu_{jt}$$ (1)

where *s*_*jt*_ is the share of the population having attended educational stage *j* in year *t*. The coefficient *β*_*j*_ in this case estimates the (linear) time trend in the (logistically transformed) share of the population attending the given educational stage. The model estimates country-specific constants (*α*_*j*_) as well as separate coefficients *β*_*j*_ for each country (random slopes in a multi-level model where the two levels are countries and years, *μ*_*jt*_ constitutes the error term), so that the predicted educational expansion over time is allowed to follow different trajectories across countries. In this model, countries are expected to eventually approach a 100% attendance rate for primary and secondary education. It appears unrealistic to expect countries to converge to a 100% attendance rate of tertiary education. We therefore arbitrarily set a ceiling at 70% for tertiary education. As shown later, predictions using this ceiling fit the data well.

The coefficients from these models are used to project attendance of educational stages for the period 2015–2040. In order to arrive at the seven educational categories of the historical data, we multiplied attendance shares with the completion rates observed in 2010 for each given country and gender (we multiplied the share of the population that is predicted to attend an educational stage in year *t*, by ‘share completed in 2010’ / ‘share attended in 2010’). In a small set of cases, predicted levels of attending secondary education exceeded predicted levels of primary education attainment by a small margin. To assure that the shares of educational categories eventually summed up to 1 we set attendance of primary education to the level of secondary education attendance in those cases.

To safeguard comparability across historical time periods and projections, we decided to present predicted results based on our growth models for all the time period 1950–2040. In the results section we examine the fit of our predicted numbers to actual numbers, which appears to be highly accurate.

## Methods

In this paper, we treat educational attainment as an *ordinal* variable. This is a non-trivial decision with important implications that stands in sharp contrast to previous studies on education variability across individuals. Studies like Castelló and Domenech [21], Benaabdelaali et al. [22] or Jordá and Alonso [25] cardinalize the Barro and Lee dataset using different techniques (the first two estimate the average length of each education cycle while the last one fits a continuous distribution to capture ‘within-cycle variations’). Others, like Meschi and Scervini [23] or Morrisson and Murtin [1, 24], work with the cardinal variable ‘years of schooling’. There are several reasons why we have decided to treat education as an ordinal variable. On the one hand, cardinal variables like ‘years of schooling’ can be a poor proxy of the substantive type of education individuals might have received and their interpretation can be biased because of (i) the country-specific duration of different education cycles, and (ii) the existence of repeaters. In addition, ‘years of schooling’ is quite prone to measurement error due to recall bias. On the other hand, ordinal variables are much less prone to measurement error. As opposed to what happens with ‘years of schooling’, the meaning of the ordinal variable categories (e.g. attaining primary, secondary or tertiary education) is reasonably comparable across countries and over time.

The major disadvantage of using ordinal variables is that their variability cannot be ascertained with well-known measures like the standard deviation, the Gini coefficient or the Theil index, among many others (indeed this is *the* key methodological reason why education variability studies have always relied on cardinal variables). One of the contributions of this paper is to partially fill this gap and to enlarge the practitioner’s toolkit by proposing a new variability measure specifically designed for ordinal variables.

### A new measure of education variability for ordinal data

The tools available to assess variability are substantially reduced when working with ordinal variables (the main reason being that the notion of ‘mean’–which is crucial in the definition of cardinal measures–is not meaningful in the ordinal case; see [31]). In this section we propose a new measure of variability for ordinal variables that has useful decomposability properties. Let *k* be the generic number of education categories we will be working with (in our case *k* = 7) and let *N* be the population size. We will denote the number of individuals in the population with educational attainment *i* (with 1 ≤ *i* ≤ *k*) as *N*_*i*_, and *p*_*i*_ = *N*_*i*_/*N* will be the corresponding population share. We define our ordinal variability measure as

$$V(p_{1},\cdots ,p_{k})≔\sum_{i=1}^{i=k}\sum_{j=1}^{j=k}p_{i}p_{j}I(i,j)$$ (2)

where I(i,j) is an indicator function that takes a value of 1 whenever *i* ≠ *j* and 0 otherwise. This measure indicates the probability that two randomly chosen individuals have *different* education attainments. Whenever all individuals have the same educational achievement (i.e. *p*_*i*_ = 1 for some category *i*) there is no variability, so *V* = 0. For any other distribution, *V* takes strictly positive values, and the higher the values, the more disperse the corresponding education distribution is.

A useful feature of the ordinal variability index suggested here is that it is nicely decomposable when the population is partitioned into different groups. In this paper we consider the partition of the population between women and men (*N*^*f*^ and *N*^*m*^ denote their corresponding population sizes), but any other population partition in an arbitrary large number of groups would do as well. Let $N_{i}^{f},N_{i}^{m}$ be the number of women and men with educational attainment *i* (hence $N_{i}^{f}+N_{i}^{m}=N_{i}$). Their relative shares are denoted as $p_{i}^{f},p_{i}^{m}$. It is straightforward to check that our variability index can be decomposed as

$$V=s_{f}V_{f}+s_{m}V_{m}+s_{b}(A_{f}+A_{m})$$ (3)

where

$$V_{f}=V(p_{1}^{f},\cdots ,p_{k}^{f})$$ (4)

$$V_{m}=V(p_{1}^{m},\cdots ,p_{k}^{m})$$ (5)

$$A_{f}=\sum_{i=2}^{i=k}\sum_{j<i}p_{i}^{f}p_{j}^{m}$$ (6)

$$A_{m}=\sum_{i=2}^{i=k}\sum_{j<i}p_{i}^{m}p_{j}^{f}$$ (7)

$$s_{f}={\left(N^{f}/N\right)}^{2}$$ (8)

$$s_{m}={\left(N^{m}/N\right)}^{2}$$ (9)

$$s_{b}=2N^{f}N^{m}/N^{2}$$ (10)

The derivation of Eq (3) is shown in the S1 Appendix. *V*_*f*_ and *V*_*m*_ measure the degree of education variability within the groups of women and men respectively, and *s*_*f*_, *s*_*m*_, *s*_*b*_ represent the relative weight of each component depending on the population size of each group. The “between-group component” *A*_*f*_ (resp. *A*_*m*_) measures the probability that a randomly selected woman has higher (resp. lower) educational attainment than a randomly selected man. Therefore, *A*_*f*_ and *A*_*m*_ can be interpreted as women’s educational advantage over men and vice versa. Interestingly, the ideas discussed in Herrero and Villar [32] bear some similarity with the measures proposed in this paper. Yet, the objectives of the two papers are entirely different: while Herrero and Villar [32] ultimate aim is to evaluate and rank groups’ performance in an ordinal setting, our aim is to decompose ordinal variability in four meaningful components. Finally, we define the extent of gender (in)equality in a given society as *G*: = *A*_*f*_ − *A*_*m*_, a measure ranging from −1 to 1. If *G* = −1, there is no woman whose educational attainment is higher than that of any man, and if *G* = 1, the reverse is true. When the education distributions of women and men are identical, *G* = 0. This measure is not monotonic: values above 0 reflect a better state of affairs for women and vice-versa. The additive decomposition formula shown in (3) articulates into a coherent whole overall education inequality and its four basic subcomponents.

### Variability versus inequality

It should be noted that the index presented in Eq (3) is a measure of ‘variability’ or ‘dispersion’, but *not* of ‘inequality’. Both concepts are highly related, but there is a subtle difference between them. While variability or dispersion measures like the standard deviation simply describe the spread of a given distribution, inequality measures involve value judgements regarding equity and can be made more sensitive to changes occurring in different parts of the distribution (see [33]). Even if such sensitivity might make inequality measures more appealing at first sight, other practical considerations make them less attractive. Most importantly, in the ordinal setting considered in this paper existing inequality measures cannot be broken down in a way analogous to Eq (3). Unlike their cardinal counterparts, current measures of ordinal inequality do *not* admit additive decompositions into a within-group and a between-group component. Since such decomposition is crucial to address the research questions of this paper, we adhere to the notion of ‘variability’ when documenting the ways in which education is distributed across individuals.

Previous studies investigating the distribution of education over time have relied as well on variability / dispersion measures (e.g. Dorius [3]). For the sake of completeness, we have compared our results with the ones we would obtain using the ordinal inequality measures proposed by Abul Naga and Yalcin [34] and Kobus and Milos [35]. It turns out that they are highly correlated (r = 0.88; details not shown here but available upon request).

Interestingly, the variability measure proposed in this paper has much in common with other classical measures of heterogeneity defined in the context of nominal and cardinal variables respectively: the ‘index of fractionalization’ and the ‘Gini coefficient’. Indeed, all three measures are extremely similar because they are grounded in the same basic principle: two individuals are picked at random and one inspects whether (or to what extent) they share a given characteristic/attribute. The only difference between the nominal, ordinal and cardinal cases is the metric that is used to assess the similarity between pairs of individuals. In the nominal case one inspects whether the two individuals belong to different pre-specified groups or not, in the ordinal one whether one of the corresponding attainments is superior to the other or not and in the cardinal case one takes into account the distance between the corresponding (cardinal) attainments. Not surprisingly, all three measures have an extremely similar functional form. The fractionalization index for nominal variables can be written exactly as in Eq (2) and the Gini coefficient can be written as

$$\sum_{i=1}^{i=k}\sum_{j=1}^{j=k}p_{i}p_{j}|\left(y_{i}/\mu )-(y_{j}/\mu \right)|$$ (11)

where *y*_*i*_ is the value of the cardinal variable one is interested in for group *i* and *μ* is the mean of the distribution. In this respect, our index *V* can be thought as the ‘missing link’ between the index of fractionalization and the Gini coefficient.

### Interpreting variability decompositions

Eq (3) is reminiscent of well-known additive decompositions of inequality in within- and between-group components for the cardinal case (e.g. like the ones used in Generalized Entropy measures, like the Theil index or the Mean Log Deviation). Yet, the interpretations in both cases are entirely different. In the cardinal case, the between-group component is the inequality that would be observed in a hypothetical distribution where each individual had the same educational attainment as the *mean* in his group. Yet, it does not make sense to speak about the contribution of between-group variability to overall variability in the ordinal context because the notion of ‘mean’ is not applicable. Instead, the term *s*_*b*_(*A*_*f*_ + *A*_*m*_) in Eq (3) should be interpreted as the proportion of pairwise comparisons where individuals have different educational attainments involving a woman and a man. To illustrate the difference between both approaches consider a hypothetical scenario where the number of women and men and their educational attainments turned out to be exactly the same. For the cardinal case the between-group component would go to zero because the gender-specific means would be the same. In the ordinal setting proposed here, the between-group component would amount to 50% because half of the education comparisons between pairs of randomly selected individuals would involve a woman and a man. Indeed, in such a hypothetical scenario the contribution to overall variability of the four subcomponents shown in (3) would be exactly the same: 25%. These fundamental differences should be taken into account when interpreting the results.

## Empirical findings

In this section, we present our empirical findings based on the systematic exploration of the seven education categories’ distributions reported in the Barro and Lee [7] dataset. We start documenting the global education expansion during the period 1950–2040 using our model predicted data (see section 3). Afterwards, we calculate the education variability index (together with its four subcomponents) and the gender gap in education. We report their values and trends–both globally and regionally–and quantify the contribution of the different subcomponents to overall education variability. Finally, we analyze the relationship between overall education variability and the gender gap in education.

### Education expansion

**Fig 3:**
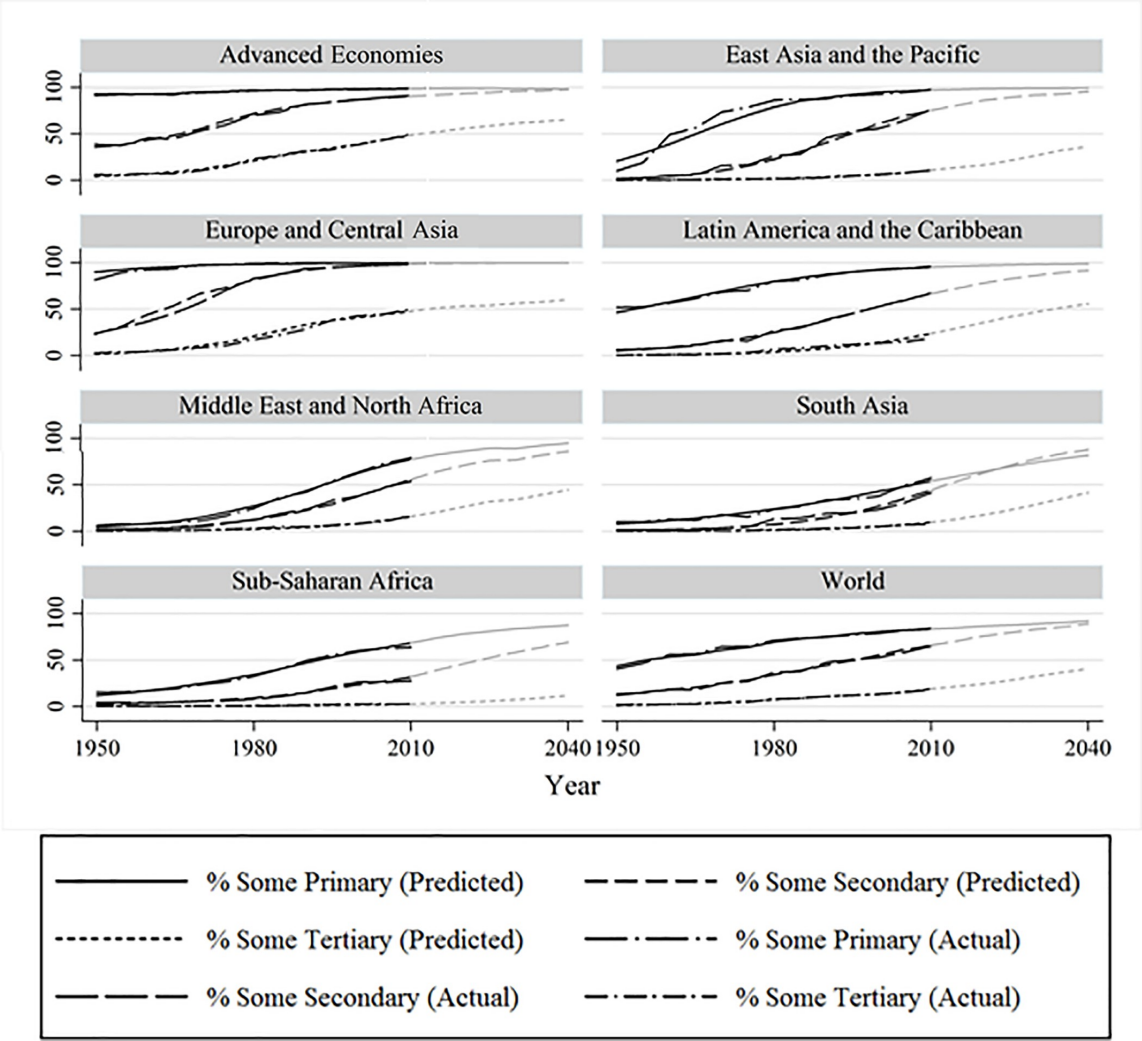
Women’s attendance of educational stages 1950–2040, by region, predicted and actual (weighted by population size of countries). Parts in greyscale are predicted values. Source: Authors’ calculations based on the BL dataset.

Fig 3 presents the share of women having attended primary, secondary, and tertiary education for seven world regions as well as the predicted shares based on our models. Whereas attendance to primary education was close to being universal in Advanced Economies and Eastern Europe in 1950, shares close to universality have now also reached East Asia and Latin America, and this pattern is expected to extend to South Asia, the Middle East and Africa by 2040. Attendance to secondary education was still a minority phenomenon in all world regions in 1950, but today a majority of individuals in most world regions attends secondary education. In all regions but South Asia and Sub-Saharan Africa more than 90% of women are expected to attend secondary education by 2040. Tertiary education was not very extended in the 1950s, but almost half of the women aged 30–34 are expected to attain tertiary education by 2040 in all regions but Sub-Saharan Africa, where tertiary education is predicted to increase only slowly. Figures for men are similar and displayed in S1 Fig of the S1 Appendix.

For the period 1950–2010, Fig 3 shows both predicted and actual shares of attendance. The fact that the two sets of shares are hardly distinguishable from each other in the graph reflects the good fit of the logistic growth curve models described in section 3. Only for secondary education in Eastern Europe and primary education in East Asia some differences between both become visible in certain time periods. Due to this relatively neat fit between predictions and reality, we believe that presenting our model-predicted results does not imply a qualitatively important loss of information.

### Evolution of overall education variability and its components

**Fig 4:**
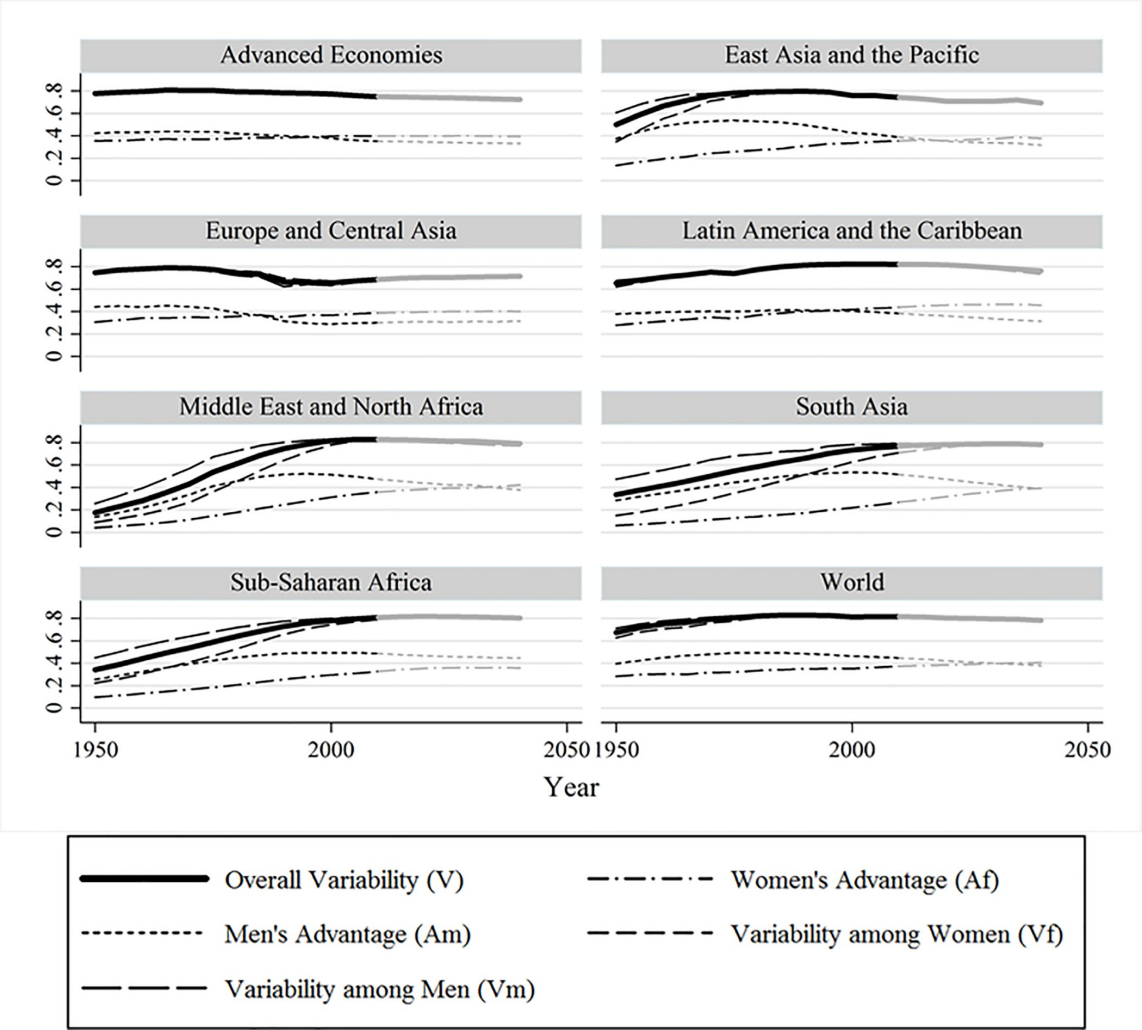
Education variability and its components across time and space, 1950–2040, weighted by country population size. Parts in greyscale are predicted values. Source: Authors’ calculations based on the BL dataset.

Fig 4 displays the development of education variability across the world and within world regions based on ordinal educational categories. It also simultaneously displays the four components of the variability decomposition formula shown in Eq (3). The thick solid line represents overall variability *V* over time and roughly follows an inverted U-shaped curve worldwide, congruent with the results of Dorius [3] and Morrisson and Murtin [24]. Most regions individually appear to follow parts of that curve during the period under study. In some regions variability used to be still relatively low in the 1950s, but dispersion increased with time and educational expansion. In other regions, variability was already high but has recently started to decrease slightly. This decrease is expected to continue in the future, and to find its expression in a decline in educational variability worldwide. The region of Eastern Europe and Central Asia seems to follow a slightly different trend. While we also observe an inverted U-shaped trajectory until the 1990s, from that year onwards education variability seems to take off and increase until 2010 (with further increases expected until 2040)–a trend that might be partly attributable to the collapse of the communist bloc and the swift transition to capitalist economies.

In each of the panels in Fig 4 we also show education variability levels among women and men (i.e. *V*_*f*_ and *V*_*m*_). The dashed curves for variability within men and women are only visible during the period 1950–2000 and only for those regions with lower levels of education (i.e. the regions of Sub-Saharan Africa, the Middle East and North Africa, East Asia Pacific and South Asia). In those regions, variability in terms of education among women was much lower than variability among men in the 1950s. By the 2010s, variability among men and women became very similar to overall education variability, so the three curves are indistinguishable from each other in the right tails of the graphs. This means that in the past women used to be a considerably more homogeneous group in terms of education compared to men. Surprisingly, despite the observed reversal in the gender gap and its expected persistence in the future, men and women are expected to exhibit the same level of education variability during the period 2010–2040. The educational disadvantage of men therefore does not seem to translate into them being a more homogeneous group in terms of education compared to women–as we were originally expecting. Overall, this suggests that the stylized trajectories shown in Fig 1 are a rough approximation of what has actually happened with overall and gender-specific education variability in the different world regions.

Lastly, the panels in Fig 4 also show the values of *A*_*f*_ and *A*_*m*_ (i.e. the likelihood that women are more highly educated than men and vice-versa) over time. While the relative position of these curves varies across regions, they roughly follow a similar pattern: *A*_*m*_ increases first and at some point it starts declining, while *A*_*f*_ increases unabated during the whole period under consideration. Hence, the stylized trajectories shown in Fig 2 synthesize in a fairly accurate way the real patterns of female and male education (dis)advantage observed in the world and its regions. Interestingly, we observe that in most regions the two curves either cross before 2010 or are expected to do so between 2010 and 2040 (Sub-Saharan Africa is the only exception), thus meaning that the gender gap reversal has already occurred or is expected to occur in the coming decades–an issue to which we now turn.

### Gender gap reversal in education

**Fig 5:**
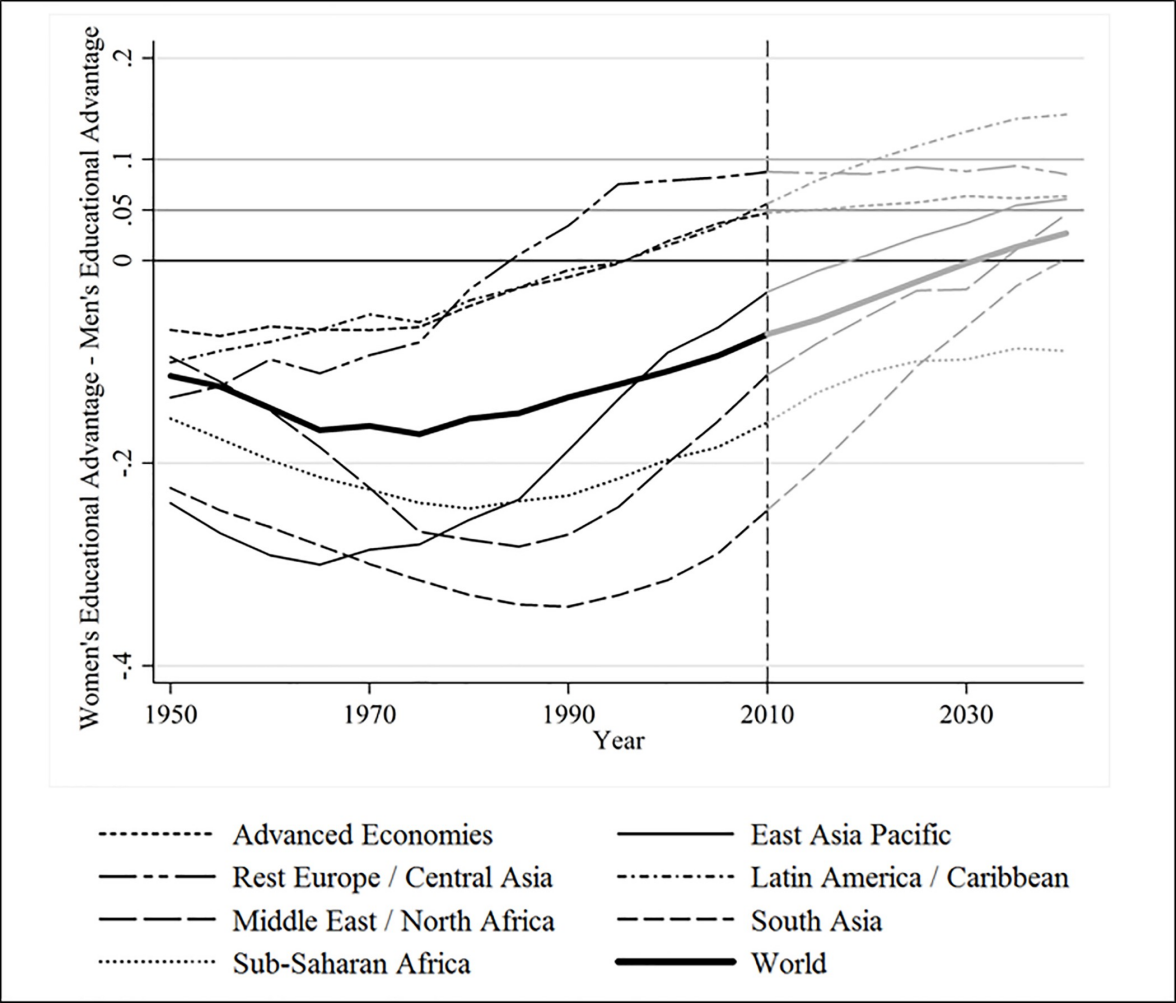
Gender Gap in Education across the period 1950–2040, weighted by country population size. Parts in greyscale are predicted values. Source: Authors’ calculations based on the BL dataset.

Fig 5 displays, for the seven world regions as well as for the world overall, how the gender gap in education has actually developed from 1950 to 2010 and how it is expected to develop across time until 2040. Recall that negative values represent distributions where women are likely to be less educated than men and vice versa. In line with previous research, one can observe that women used to be less educated than men in the past in all world regions. But, this gender gap has reduced dramatically across the globe, and even reversed in the Advanced Economies, Eastern Europe, and Latin America well before 2010. The gender gap in these three regions must have reached its historical minimum in a period preceding 1950. The evolution of the gender gap has not always been monotonic: in the regions of Sub-Saharan Africa, the Middle East and North Africa, East Asia Pacific and South Asia it has initially *declined*, attained its minimum somewhere between 1960 and 1990 and then started increasing towards gender equality. By 2040, the gender gap is expected to be closed and slightly reversed in almost all world regions (the only exception being Sub-Saharan Africa). While at the beginning of our observation window we can clearly distinguish two clusters of regions (on the one hand the Advanced Economies, Eastern Europe, and Latin America–henceforth referred to as ‘forerunning regions’–and on the other hand Sub-Saharan Africa, the Middle East and North Africa, East Asia Pacific and South Asia–referred to as ‘laggard regions’) almost all gender gaps are expected to converge towards a slightly positive value indicating some educational advantage in favor of women by 2040. Inspecting the ‘global gender gap in education’ (i.e. taking the entire world as a unit of analysis), we also observe a U-shaped trajectory over time. In 2010, the global gender gap was still in favor of men but this is not expected to be the case anymore by 2040. This story differs from the conclusion of an ever-decreasing gap in education between men and women over time once taking a relative measure of educational differences (e.g. [7] Chapter 2.6).

### Contribution of the different components to overall education variability

Table 1 displays the relative contribution of each of the four components discussed in the previous section to overall education variability (these are derived from decomposition formula (3)). Recall that, as a reference, in a hypothetical country where the educational attainment of men was identical to that of women, the percent contribution of the four components shown in Table 1 would be exactly the same: 25%. As can be seen, the composition of education variability has been shifting dramatically over time. Back in the 1950s, variability among women contributed very little to overall education variability in the laggard regions (e.g. a mere contribution of 9.9% in South Asia), while the opposite was true for variability among men (e.g. 38.9% in the same region). At the beginning of our observation period, the educational advantage of men over women was by far the main contributor to education variability in the world as a whole and in most of its regions (see last column in Table 1). Sixty years later, that contribution has decreased substantially at a global level. Indeed, in the forerunning regions (i.e. in most high- and middle-income countries) it has become the least important contributor to education variability (i.e. 23.5%, 21.9% and 23.3% for the advanced economies, Eastern Europe and Latin America in 2010). Concomitant with these changes, the educational advantage of women over men has become an increasingly important ingredient of overall education variability (see second-to-last column in Table 1). Sixty years ago, the contribution of that kind of variability to overall education variability was by far the *least* important among the four (particularly so in the poorest regions of the world, e.g. only 9% in South Asia). Nowadays, women’s educational advantage is the main contributor to education variability in most high- and middle-income countries (i.e. 26.6%, 28.3% and 26.7% for the advanced economies, Eastern Europe and Latin America in 2010).

**Table 1:** Variability in educational attainment by region and year for 25–29 year-olds, decomposed into within and between women/men components.

Region	Year	%C*V*_*f*_	%C*V*_*m*_	%C*A*_*f*_	%C*A*_*m*_
Advanced Economies	1950	26.8	23.2	22.8	27.2
Advanced Economies	1980	24.5	25.4	23.6	26.5
Advanced Economies	2010	24.4	25.6	26.6	23.5
Advanced Economies	2040	23.7	26.2	27.2	22.8
East Asia and the Pacific	1950	16.1	32.9	13.5	37.5
East Asia and the Pacific	1980	22.8	26.6	17.2	33.4
East Asia and the Pacific	2010	24.2	25.7	24.0	26.1
East Asia and the Pacific	2040	21.7	28.2	27.2	22.8
E. Europe & Central Asia	1950	33.1	17.8	20.1	28.9
E. Europe & Central Asia	1980	25.6	24.3	24.1	26.0
E. Europe & Central Asia	2010	25.8	24.0	28.3	21.9
E. Europe & Central Asia	2040	23.8	26.1	28.1	22.1
Latin America & Carib.	1950	23.7	26.0	21.3	29.0
Latin America & Carib.	1980	25.1	24.8	23.7	26.3
Latin America & Carib.	2010	25.7	24.2	26.7	23.3
Latin America & Carib.	2040	23.4	26.0	30.0	20.6
Middle E.& North Africa	1950	12.1	37.4	11.7	38.7
Middle E.& North Africa	1980	18.6	29.6	14.6	37.2
Middle E.& North Africa	2010	23.2	26.5	21.7	28.5
Middle E.& North Africa	2040	22.9	26.7	26.6	23.7
South Asia	1950	9.9	38.9	9.0	42.2
South Asia	1980	16.0	31.8	12.0	40.2
South Asia	2010	21.7	27.4	17.5	33.5
South Asia	2040	22.3	27.7	25.0	24.9
Sub-Saharan Africa	1950	16.6	31.9	14.4	37.1
Sub-Saharan Africa	1980	21.2	27.4	16.1	35.3
Sub-Saharan Africa	2010	23.7	26.0	20.2	30.1
Sub-Saharan Africa	2040	24.7	25.2	22.3	27.8
World	1950	23.2	26.5	20.9	29.4
World	1980	23.6	26.1	20.4	29.9
World	2010	24.4	25.5	22.8	27.3
World	2040	23.1	26.9	25.9	24.1

*V*: Overall variability; *V*_*f*_, *V*_*m*_: Variability among women and men respectively; *A*_*f*_/*A*_*m*_: Probability that a randomly selected woman/man is more educated than a randomly selected man/woman; %C: Percent contribution of the different components. Authors’ calculations based on Barro and Lee (2013)[7].

### The relationship between gender equality and overall variability

**Fig 6:**
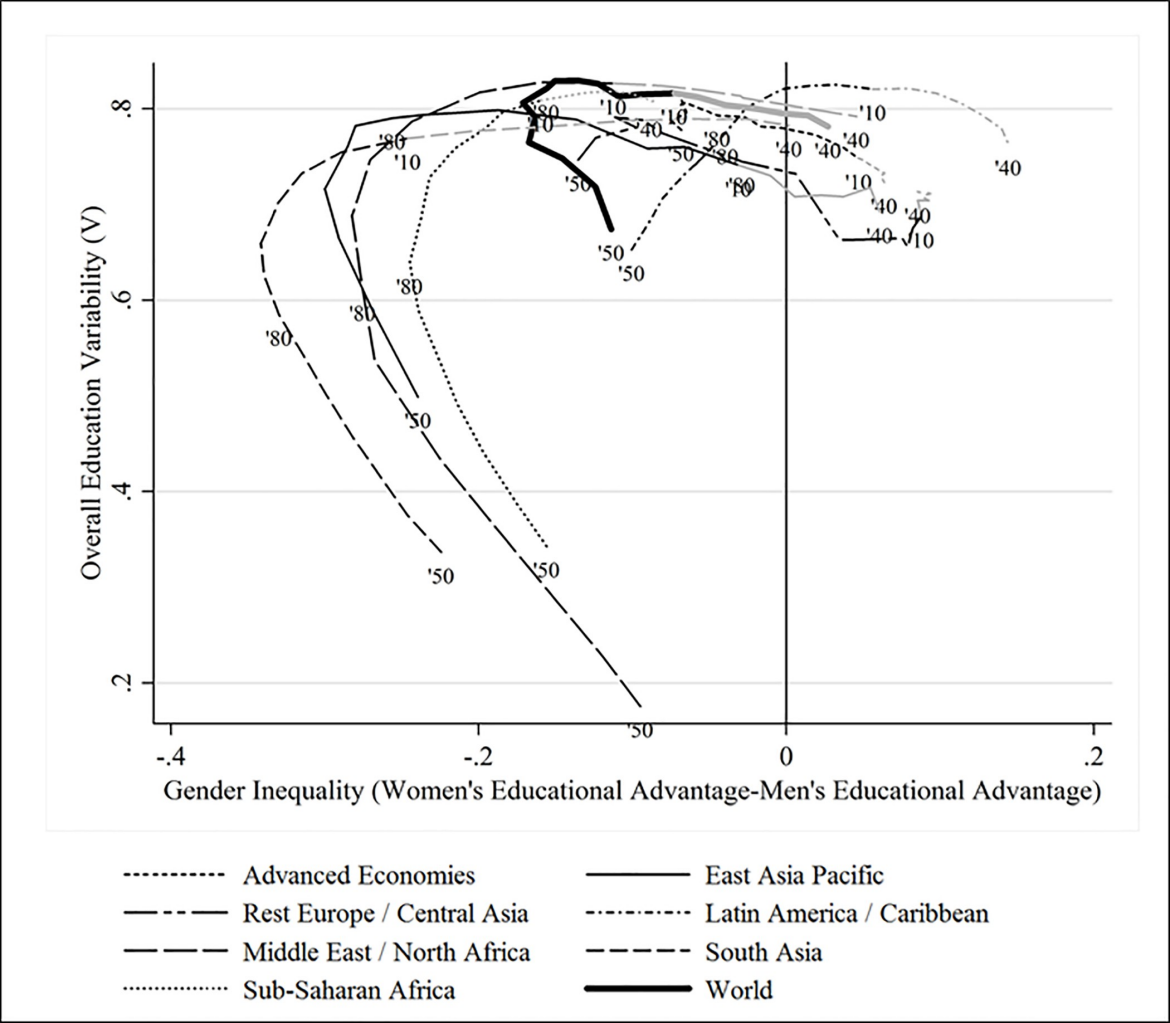
Development of gender and overall variability in education over time. **Parts in greyscale are predicted values.** Source: Authors’ calculations based on the BL dataset.

What can we say about the relationship between the gender gap in education and overall education variability in the midst of the aforementioned compositional changes? Have they moved in the same or in opposite directions? To give a precise account of the joint levels and trends of *V* and *G*, in Fig 6 we present a full-fledged description of how these two indicators have co-varied over time for the world and its regions (the values of *V* are shown in the vertical axis and those of *G* in the horizontal one). Putting together the world regions’ experiences shown in that figure, we conclude this section presenting a broad-strokes account of the joint evolution of education and gender inequality, which consists of four stages. Stage I: The male-dominated education expansion brings increases in education variability and drives the gender gap in education in favor of men. In this case, both measures of variability temporarily go in the normatively undesirable direction, and might therefore be a period where educational expansion has most externalities. Stage II: The delayed incorporation of women into mass education tilts the gender gap towards the opposite direction and brings further increases in education variability until it reaches its maximum. Here, both measures of variability go in opposite directions and trade-offs between both policy objectives might emerge. During this stage potential efforts to slow down education expansion in order to prevent overall education variability from rising further could be at odds with reducing the gender gap in education. Stage III: When further education expansion gradually shifts the population towards the higher educational categories, education variability starts declining and the gender gap goes to zero: this is the onset of a period where education expands while both measures of variability decrease simultaneously. Stage IV: Further education expansion, particularly in favor of women, reverses the gender gap in education while further decreasing overall education variability.

Even if not all regions (let alone the individual countries) fit this stylized description, it reasonably represents the different trajectories shown in Fig 6. For the laggard regions of the Middle East and North Africa, East Asia Pacific and South Asia we can observe the occurrence of stages I, II and III. Until 2010 Sub-Saharan Africa had only completed stages I and II. It is expected that by 2040 Sub-Saharan Africa will have entered the third stage. As regards the advanced economies and Eastern Europe, we observe the occurrence of stages II, III and IV during our 1950–2040 study period (in S2 Fig of the S1 Appendix we show how many years each region has spent in each of the aforementioned stages). Lastly, the region of Latin America stands out as being the only one that does not fit very well our 4-stage narrative. The initial reductions of the gender gap go in tandem with increases in education variability and the posterior decreases in education variability occur when the gender gap reverses in favor of women (i.e. both measures tend to go in opposite directions). Whereas future educational expansion in other regions is expected to simultaneously reduce both forms of heterogeneity, expansion in Latin America is likely to increase the gender gap favoring women to relatively high levels.

## Summary and concluding remarks

**Fig 7:**
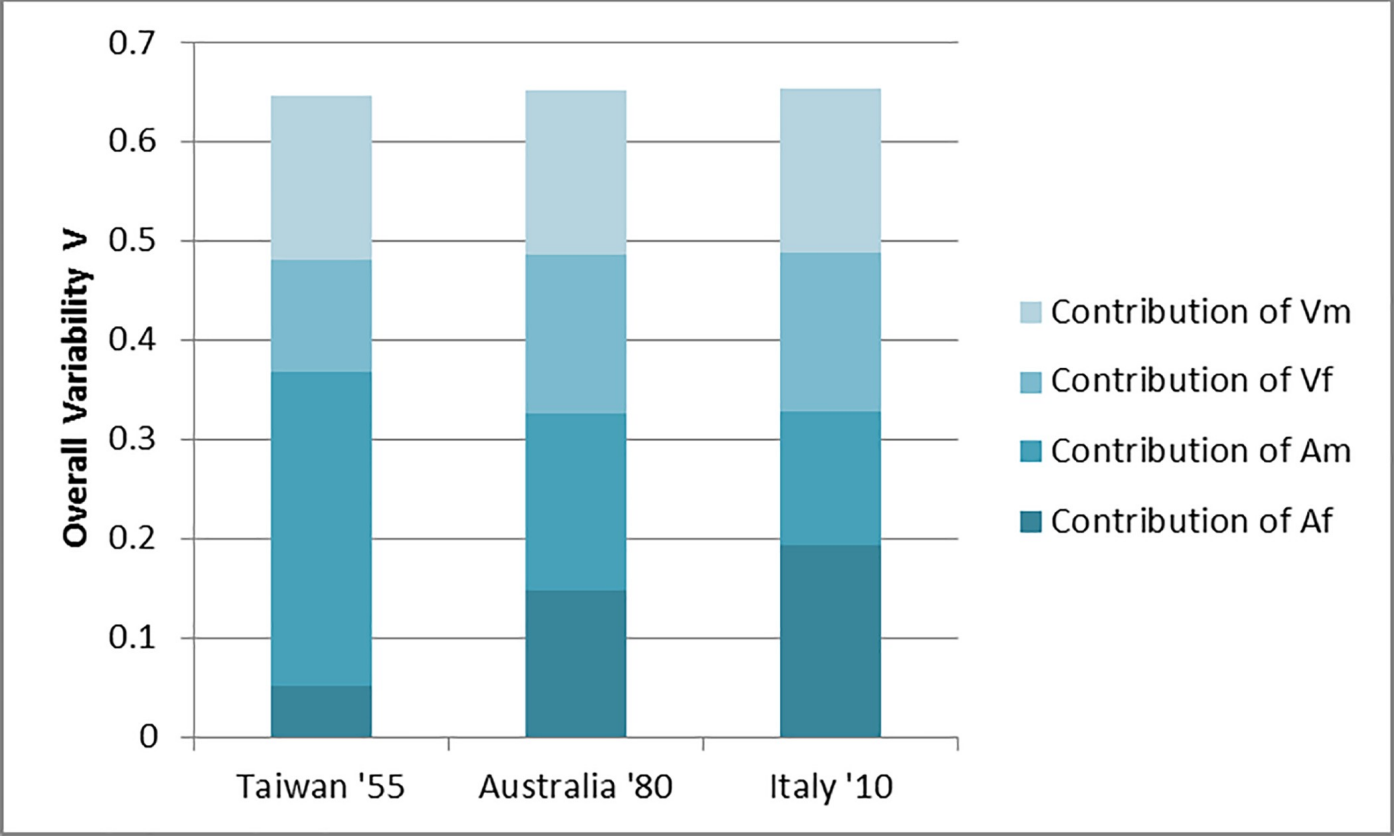
Education variability and its decomposition in four components for Taiwan (1955), Australia (1980) and Italy (2010). Source: Authors’ calculations based on the BL dataset.

In this paper, we have documented the trends in education variability for the world and its regions. In addition, we have presented a new technique that allows decomposing overall education variability in four clearly interpretable components: variability among women, variability among men, educational advantage of men and educational advantage of women. For that purpose, we have developed a new variability index adapted to the ordinal nature of educational attainment, which for the first time allows articulating the aforementioned components into a coherent whole. Such decomposition techniques are extremely useful to go beyond mere descriptions of levels and trends. They allow pinpointing not only what are the most important factors that explain current education variability levels, but also quantify their contribution to changes over time. As an illustration, Fig 7 shows the decomposition of education variability for three countries: Taiwan (for the year 1955), Australia (1980) and Italy (2010). The three countries have very similar levels of overall education variability. Yet, the decomposition of this measure yields completely different results. While in Taiwan (1955) most of the variability can be attributed to the educational advantage of men over women (a component explaining 31.7% of the total), the reverse happens in Italy (2010). There, the educational advantage of men over women and of women over men are the least and most important contributors to overall education variability, respectively (these components explain 13.4% and 19.4% of the total). Somewhere in between we have the case of Australia (1980), where the four components explain about the same percentage of education variability. The possibility of breaking down outcome indicators into meaningful and policy-relevant pieces of information can be very useful for the design, implementation and evaluation of fine-tuned education programs.

Based on the Barro and Lee [7] dataset we have calculated the values of the variability index and the contribution of its components for 146 countries during a period spanning almost a century (1950–2040). Our findings indicate that the composition of education variability has changed dramatically during the period between 1950 and 2010. In the past, women used to be a much more homogeneous group compared to men in terms of education in several world regions. With education expansion, variability among women quickly started to increase. We expected that as the gender gap in education reversed in favor of women, the variability among them would be higher than the variability among men. Surprisingly, our results show that education variability among men and women is now very similar in all world regions, and this is expected to remain so in the near future. Back in the 1950s, the educational advantage of men over women was by far the main contributor to education variability in the world as a whole and in most of its regions, while the opposite was true about the educational advantage of women over men. Nowadays, men’s educational advantage has become the least important contributor to education variability in several regions, while women’s educational advantage is the main contributor to education variability in most high- and middle-income countries.

Another of the issues investigated in this paper has been the relationship between education variability and the gender gap in education. Our findings suggest that during the process of education expansion, most world regions have gone (or are expected to go) through the following consecutive phases: (I) increasing education variability and gender gap in education in favor of men; (II) increasing education variability but reduction in the gender gap in education; (III) reduction of education variability and the gender gap in education; and (IV) stalling or slight reduction of education variability and reversal of the gender gap in education in favor of women. One could speculate that when societies enter the third phase, an opportunity window opens up where educational expansion might bring the additional benefits of reducing both overall and gender inequality in education (i.e. a period where there is no dilemma between ‘efficiency’ and both forms of ‘equality’). According to our results, most world regions have experienced, are experiencing or are expected to experience a period whereby both education variability and the gender gap decline simultaneously. Whether or not the confluence of these phenomena is truly beneficial for the countries that experience them is a matter for future research. In addition, if one assumes that large educational advantages of one sex over the other are normatively undesirable, our findings suggest that in the near future there might be some trade-offs between gender and overall education variability, as they both seem to run in opposite directions. If further education expansion contributes to further decreases in overall education variability *and* increases in females’ educational advantage over men (a scenario that appears likely until a ceiling of tertiary education attainment is reached in many countries), education planners might be facing ethical dilemmas upon which it will be necessary to reflect (particularly in high-income countries).

What should one expect for the more distant future? Will education variability continue to decline and will the gender gap increasingly favor women? In line with Inglehart and Welzel [36], our expectations are that as long as the modernization process continues to unfold, it will facilitate cultural changes that make gender equality increasingly likely. Since we speculate that gender equality has come to stay, we expect that the gender gap in education will not continue increasing to attain the high levels it had in the initial stages of the male-dominated education expansion, but will rather hover around gender parity levels.

As regards education variability overall there are a couple of reasons to *not* expect a continuation of its currently downward trend for a long time. First, while the global decline in overall education variability (both for women and for men) points to an increasing equalization of opportunities across citizens worldwide, it is likely that new forms of education variability not captured in our data might be (or already have been) replacing older ones. Indeed one can suspect that the observed declines in overall education variability can be attributed to the clustering of the educational attainment distribution at its top, which might be hiding an increasing diversity of superior education alternatives (like Masters and PhDs) not captured in the seven categories of Barro and Lee’s dataset. Some exploratory work carried out for the case of the US using census microdata samples (not shown here but available upon request) suggests that this might be indeed the case. When we enlarge the set of education categories from seven to nine (including Masters and PhDs), the decline in overall education variability is less pronounced. In the same line, a recent study by Meschi and Scervini [23] suggests that after a long period of sustained decline, education inequality is turning upwards in many European countries because of the expansion of tertiary and post-tertiary schooling.

Second, education variability is intimately associated with technological progress and economic inequality, two forces that are unlikely to remain stable over time. Under the assumption that technology is skill-biased [37], technological progress will widen inequality among skill groups unless it is countered by increases in the supply of educated workers. Depending on the outcome of the ‘race between education and technology’ [38]–which is particularly uncertain–income and education inequality levels can vary to a great extent. Along similar lines, Milanovic [39] recently suggested that the modern historical era from the preindustrial through the postindustrial period is characterized by so-called ‘Kuznets waves’ of alternating increases and decreases in economic inequality, with many high-income countries currently in the upward portion of the wave. In all likelihood, the ‘economic Kuznets waves’ will translate into ‘education Kuznets waves’, thereby increasing education variability as well. Whether or not such incipient trends will consolidate in the near future is a matter for future research.

We conclude with a word of caution about the limits of our approach. Documenting the variability and decompositions of educational attainment across world countries is an important endeavor in and of itself. There is an extensive literature linking educational attainment with all kinds of normatively desirable outcomes that social scientists and policy makers are interested in–including, but not limited to, job opportunities, earning potential, health outcomes and life satisfaction across the life cycle. Yet, educational attainment is not only about quantity, but also about quality. Very often, students do not acquire the skills they need by merely attending low quality schools with insufficient resources [40]. In future research, it will be necessary to complement our findings with other indicators (like test scores) that are better suited to capture the cognitive abilities and skills that students acquire while at school.

## Supporting information

S1 Appendix Derivation of Eq (3). (DOCX)

S1 Fig Men’s attendance of educational stages 1950–2040, by region, predicted and actual. (DOCX)

S2 Fig Periods during which trends in education variability and the gender gap in education go in simultaneous or different directions. (DOCX)
