# Supplementary material for: A century of change in global education variability and gender differences in education
Source: PLoS One. 2019 Feb 27;14(2):e0212692. doi: 10.1371/journal.pone.0212692 (PMC6392467; doi:10.1371/journal.pone.0212692)
Supplement: S1 Appendix — (DOCX) [file pone.0212692.s001.docx]

**Supporting Information**

**S1 Appendix.** Derivation of Equation (3).

Manipulating algebraically it is easy to show that

$$V\left( p_{1},\cdots,p_{k} \right)=\sum_{i=1}^{i=k} \sum_{j=1}^{j=k} p_{i}p_{j}\mathbb{I}\left( i,j \right)=\frac{\sum_{i=1}^{k} \sum_{j\neq i} N_{i}N_{j}}{N^{2}}=\frac{\sum_{i=1}^{k} \sum_{j\neq i} (N_{i}^{f}+N_{i}^{m})(N_{j}^{f}+N_{j}^{m})}{N^{2}}$$

$$=\frac{\sum_{i=1}^{k} \sum_{j\neq i} N_{i}^{f}N_{j}^{f}+\sum_{i=1}^{k} \sum_{j\neq i} N_{i}^{m}N_{j}^{m}+\sum_{i=1}^{k} \sum_{j\neq i} N_{i}^{f}N_{j}^{m}+\sum_{i=1}^{k} \sum_{j\neq i} N_{i}^{m}N_{j}^{f}}{N^{2}}$$

$$=\left( \frac{N^{f}}{N} \right)^{2}\frac{\sum_{i=1}^{k} \sum_{j\neq i} N_{i}^{f}N_{j}^{f}}{\left( N^{f} \right)^{2}}+\left( \frac{N^{m}}{N} \right)^{2}\frac{\sum_{i=1}^{k} \sum_{j\neq i} N_{i}^{m}N_{j}^{m}}{\left( N^{m} \right)^{2}}+$$

$$+\left( 2\frac{N^{f}N^{m}}{N^{2}} \right)\frac{\sum_{i=1}^{k} \sum_{j\neq i} N_{i}^{f}N_{j}^{m}}{N^{f}N^{m}}$$

$$=s_{f}V_{f}+s_{m}V_{m}+\frac{s_{b}}{N^{f}N^{m}}\left( \sum_{i=2}^{i=k} \sum_{j<i} N_{i}^{f}N_{j}^{m}+\sum_{i=2}^{i=k} \sum_{j<i} N_{i}^{m}N_{j}^{f} \right)=s_{f}V_{f}+s_{m}V_{m}+s_{b}\left( \sum_{i=2}^{i=k} \sum_{j<i} p_{i}^{f}p_{j}^{m}+\sum_{i=2}^{i=k} \sum_{j<i} p_{i}^{m}p_{j}^{f} \right)=s_{f}V_{f}+s_{m}V_{m}+s_{b}\left( A_{f}+A_{m} \right).$$

This is the decomposition we were looking for.
