# Supplementary material for: A century of change in global education variability and gender differences in education
Source: PLoS One. 2019 Feb 27;14(2):e0212692. doi: 10.1371/journal.pone.0212692 (PMC6392467; doi:10.1371/journal.pone.0212692)
Supplement: S1 Fig — (DOCX) [file pone.0212692.s002.docx]

**S1 Fig.** Men’s attendance of educational stages 1950-2040, by region, predicted and actual (weighted by population size of countries).


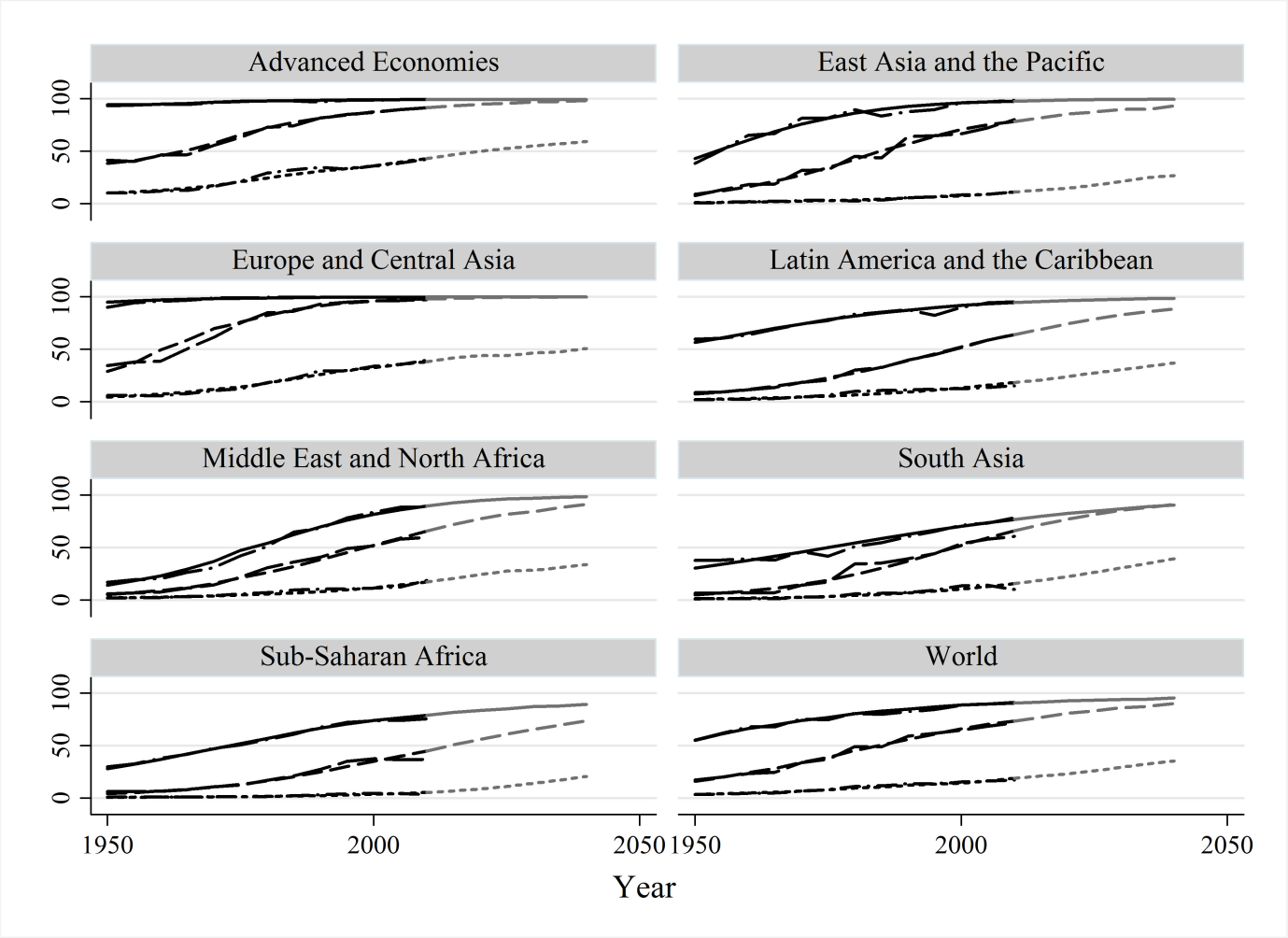


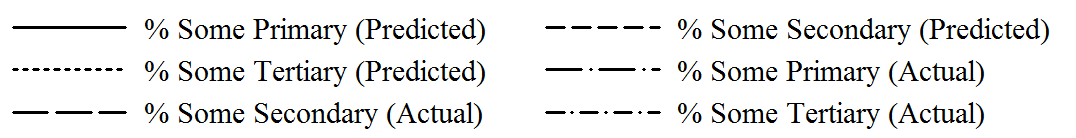


Parts in greyscale are predicted values.

Source: Authors’ calculations based on the BL dataset.
