# Supplementary material for: A century of change in global education variability and gender differences in education
Source: PLoS One. 2019 Feb 27;14(2):e0212692. doi: 10.1371/journal.pone.0212692 (PMC6392467; doi:10.1371/journal.pone.0212692)
Supplement: S2 Fig — (DOCX) [file pone.0212692.s003.docx]

**S2 Fig.** Periods during which trends in education variability and the gender gap in education go in simultaneous or different directions.

|  | 1950-1955 | 1955-1960 | 1960-1965 | 1965-1970 | 1970-1975 | 1975-1980 | 1980-1985 | 1985-1990 | 1990-1995 | 1995-2000 | 2000-2005 | 2005-2010 | 2010-2015 | 2015-2020 | 2020-2025 | 2025-2030 | 2030-2035 | 2035-2040 |
| --- | --- | --- | --- | --- | --- | --- | --- | --- | --- | --- | --- | --- | --- | --- | --- | --- | --- | --- |
| Advanced Economies | 1 | 2 | 1 | 3 | 2 | 4 | 4 | 4 | 4 | 4 | 4 | 4 | 4 | 4 | 4 | 4 | 4 | 4 |
| East Asia and the Pacific | 1 | 1 | 1 | 2 | 2 | 2 | 2 | 2 | 4 | 4 | 2 | 4 | 4 | 4 | 2 | 4 | 2 | 4 |
| Europe and Central Asia | 2 | 2 | 1 | 4 | 4 | 4 | 4 | 4 | 2 | 4 | 2 | 2 | 2 | 2 | 4 | 2 | 2 | 2 |
| Latin America and the Caribbean | 2 | 2 | 2 | 2 | 3 | 2 | 2 | 2 | 2 | 2 | 2 | 4 | 2 | 4 | 3 | 3 | 3 | 3 |
| Middle East and North Africa | 1 | 1 | 1 | 1 | 1 | 1 | 1 | 2 | 2 | 2 | 2 | 4 | 4 | 4 | 4 | 4 | 4 | 4 |
| South Asia | 1 | 1 | 1 | 1 | 1 | 1 | 1 | 1 | 2 | 2 | 2 | 2 | 2 | 2 | 2 | 2 | 4 | 4 |
| Sub-Saharan Africa | 1 | 1 | 1 | 1 | 1 | 1 | 2 | 2 | 2 | 2 | 2 | 2 | 2 | 2 | 4 | 4 | 4 | 3 |
| **World** | 1 | 1 | 1 | 2 | 1 | 2 | 2 | 2 | 4 | 4 | 2 | 2 | 4 | 4 | 4 | 4 | 4 | 4 |

|  | Education Variability and Gender Gap Increasing |
| --- | --- |
|  | Gender Gap Decreasing, Education Variability Increasing |
|  | Gender Gap Increasing, Education Variability Decreasing |
|  | Education Variability and Gender Gap Decreasing |

In Figure S2 we indicate the starting and the end of the time periods in which the world and its regions have either experienced (i) trade-offs among education variability and the gender gap in education, (ii) increases in both types of measures, or (iii) simultaneous decreases^[[1]](#footnote-1)^. During the time frame considered here all world regions (except in Latin America) have gone through periods where education expansion has not been an egalitarian process, as it simultaneously increased education variability *and* the gender gap in education. Generally, this period has been longer for the laggard regions (e.g. 40 years in South Asia) and considerably shorter for the forerunning ones. Analogously, all regions have experienced considerable periods of time with trade-offs between overall education variability and the gender gap in education (i.e. periods in which one measure could only be reduced at the expense of increasing the other). At one extreme, the advanced economies have only experienced such periods for relatively short time spells (15 years), while at the other extreme, Latin America has experienced them almost during the entire time frame (80 years). Overall, there have been many more periods in which education variability increased in tandem with gender gap reductions than periods with the opposite combination. Lastly, the world and all its regions have also gone through considerably long periods without experiencing the efficiency-equality dilemma. While some regions are expected to pass through such periods only for a decade (e.g. South Asia, between 2030 and 2040), others have gone and are expected to go through them during 65 years (e.g. advanced economies, between 1975 and 2040). The world taken as a whole entered such period in 1990, and is expected to continue until 2040 with some small interruptions.

1. To generate this table, we have assumed that values of the gender gap between 0 and 0.1 (i.e. slight education advantage in favor of women) are *not* normatively undesirable. [↑](#footnote-ref-1)
